# Virtual screening and cellular validation of dolutegravir as a BRD9 inhibitor for attenuating pyroptosis in peritoneal mesothelial cells

**DOI:** 10.1080/0886022X.2026.2644768

**Published:** 2026-04-07

**Authors:** Junfang Gai, Xiaohong Xing, Chanjuan Gong, Yanjuan Teng, Shunjie Chen, Ming Yang, Weijuan Lou

**Affiliations:** a Department of Pathology, Affiliated Hospital of Integrated Traditional Chinese and Western Medicine, Nanjing University of Chinese Medicine, Nanjing, China; b Department of Nephrology, Shanghai Changzheng Hospital, Second Affiliated Hospital of Naval Medical University, Shanghai, China; c Department of Nephrology, Sixth People’s Hospital, Shanghai Jiao Tong University, Shanghai, China; d Department of Nephrology, Shanghai Fourth People’s Hospital, School of Medicine, Tongji University, Shanghai, China

**Keywords:** Peritoneal dialysis, peritoneal fibrosis, pyroptosis, BRD9, dolutegravir

## Abstract

**Background:**

Peritoneal dialysis is an essential therapy for end-stage renal disease; however, long-term exposure to high-glucose dialysis solutions induces pyroptosis of peritoneal mesothelial cells, promoting peritoneal fibrosis and ultimately leading to technique failure. The involvement of the epigenetic regulator bromodomain-containing protein 9 (BRD9) in this process remains unclear.

**Methods:**

Structure-based virtual screening of 3,447 FDA-approved drugs from the ZINC database identified dolutegravir as a candidate BRD9 inhibitor. Direct binding and inhibitory activity were validated using cellular thermal shift assays, IC_50_ determination, and molecular docking. Under high-glucose conditions, the effects of dolutegravir on pyroptosis-related signaling and fibrosis markers in human mesothelial cells were assessed by Western blotting, ELISA, RT-qPCR, and flow cytometry.

**Results:**

Dolutegravir directly bound to and inhibited BRD9. Under high-glucose stimulation, dolutegravir markedly suppressed NLRP3 inflammasome activation, reduced caspase-1 and gasdermin D cleavage, and decreased interleukin (IL)-1β and IL-18 maturation and release. Mechanistically, BRD9 inhibition accelerated nod-like receptor protein 3 (NLRP3) mRNA degradation and attenuated NLRP3-mediated secretion of the pro-fibrotic factor transforming growth factor-beta 1, leading to downregulation of fibrosis-related markers smooth muscle alpha-actin 2 and collagen type I alpha 1.

**Conclusion:**

This study identifies dolutegravir as a novel BRD9 inhibitor and demonstrates that BRD9 is a key regulator of high glucose-induced pyroptosis and pro-fibrotic signaling in mesothelial cells *via* modulation of NLRP3 mRNA stability. These findings suggest a new therapeutic strategy for preventing peritoneal fibrosis and highlight the potential of computational drug repurposing.

## 1. Introduction

Peritoneal dialysis (PD) stands as one of the most frequently employed renal replacement therapies for patients afflicted with end-stage renal disease. In comparison to hemodialysis, PD offers superior protection for residual renal function. One of its key advantages is that it can be conveniently carried out at home, eliminating the need for anticoagulant administration. Moreover, it does not interfere with the coagulation function, making it a more suitable option for patients with hemodynamically unstable cardiovascular diseases [1]. Leveraging the high-osmotic pressure properties of the peritoneal dialysis fluid, PD facilitates substance exchange through the parietal peritoneum, which acts as a semi-permeable membrane. This process effectively removes excess water and toxins from the bloodstream. However, a significant drawback is that within one to two years, between 50% and 80% of patients undergoing PD will develop peritoneal fibrosis (PF) [2,3]. This condition ultimately leads to the loss of peritoneal function and ultrafiltration failure, thereby restricting the widespread application of peritoneal dialysis.

Pyroptosis is a distinct form of hemolytic programmed cell death [4] and plays a pivotal role in the development of peritoneal fibrosis. Research has demonstrated that blocking either Caspase − 1 or GSDMD (gasdermin D) can curtail the activation of the nod-like receptor protein 3 (NLRP3) inflammasome and the pyroptosis induced by high glucose levels [5–7]. Consequently, this helps to alleviate inflammation and improve peritoneal fibrosis. Studies have shown that when human peritoneal mesothelial cells are exposed to high-glucose dialysis fluid, cytochrome C is released [8]. This release triggers the activation of caspase and the cleavage of poly (ADP-ribose) polymerase [9], ultimately leading to pyroptosis. During this process, mitochondrial rupture, loss of mitochondrial DNA, and depletion of mitochondrial membrane potential may also occur [9].

BRD9 is a protein characterized by the presence of a bromodomain [10]. It occupies a distinct branch within the bromodomain family and has emerged as a promising therapeutic target for various diseases. Within cells, BRD9 serves as a core, stoichiometric subunit of several chromatin remodeling complexes, most notably the non-canonical BAF (ncBAF) and GLTSCR1/1L-BCL7B/C (GBAF) complexes [11,12]. For instance, it contributes to the formation of complexes with transcriptional regulatory functions [13]. Through interactions with transcription factors and other related molecules, BRD9 precisely regulates the transcription of specific genes, thereby influencing cell growth, differentiation, and metabolism. BRD9 may contribute to fibrosis through multiple mechanisms. It has the ability to regulate fibrosis-related pathways, such as the TGF-β pathway [14]. TGF-β is a critical factor in fibrosis, as it activates fibroblasts and promotes the accumulation of the extracellular matrix [15]. It is particularly worth noting that BRD9 can influence oxidative stress levels and inflammatory responses by regulating the NOX1/ROS/NF-κB signaling axis, thereby participating in the regulation of the pyroptosis process [14]. Pyroptosis, as a highly pro-inflammatory programmed cell death pattern, not only directly leads to cell membrane rupture and the release of inflammatory factors, but also activates immune cells and fibroblasts, exacerbating the fibrotic microenvironment. Our previous bioinformatics analysis and molecular docking predictions indicated that high expression of BRD9 affects the occurrence of peritoneal fibrosis, and it is also related to the NLRP3 pathway of cell pyroptosis. At the same time, the candidate drug dolutegravir has a high affinity for the bromine domain of BRD9, and the potential regulatory role of BRD9 in chromatin remodeling on the inflammatoryosome-related genes provides us with a hypothesis for inhibition: BRD9 → interfering with the key signals of pyroptosis (such as the NLRP3/caspase-1/GSDMD axis) → alleviating peritoneal injury and fibrosis.

Dolutegravir, an integrase inhibitor previously approved for HIV treatment [16], was identified as a candidate BRD9 inhibitor through computational simulation and molecular docking analyses due to its high binding affinity with the bromodomain of BRD9. Given its favorable pharmacokinetic profile and well-established safety record, dolutegravir represents a promising candidate for drug repurposing [17]. The subsequent experiments will aim to verify its potential to alleviate high glucose-induced injury and fibrosis in peritoneal mesothelial cells *via* inhibition of the BRD9-mediated pyroptosis signaling axis.

This study aims to employ virtual screening for small-molecule inhibitors of BRD9. Subsequently, cell experiments will be conducted to verify the therapeutic potential of these inhibitors in treating peritoneal fibrosis. Therefore, investigating BRD9 inhibitors, guided by drug informatics, to alleviate peritoneal fibrosis *via* the pyroptosis pathway is of immense scientific importance and clinical value.

## 2. Materials and methods

### 2.1. Patient samples and mesothelial cell isolation

To elucidate the key molecular drivers of peritoneal fibrosis, we performed transcriptomic sequencing on mesothelial cells isolated from peritoneal dialysis effluent. A total of 20 patients with end-stage renal disease undergoing peritoneal dialysis were enrolled in this study, all of whom provided informed consent. The cohort was divided into two groups: 9 patients with a dialysis duration of less than six months (short-term) and 11 patients with a dialysis duration of two years (long-term). Effluent samples were collected from the overnight dwell of Continuous Ambulatory Peritoneal Dialysis (CAPD). The samples were processed within one hour of drainage.

Mesothelial cells were isolated from the dialysis effluent *via* centrifugation at 1000 rpm for 5 min at 4 °C. The supernatant was discarded, and the cell pellet was washed twice with physiological saline. The pellet was then resuspended in culture medium supplemented with 10% FBS and seeded into 25 cm^2^ culture flasks pre-coated with 0.1% gelatin. The cells were cultured in a 37 °C incubator with 5% CO_2_. The medium was first changed after 48–72 h of initial seeding and subsequently every 72 h. Upon reaching 70–80% confluence, typically within 7–14 days, the cells were harvested for subsequent experiments.

### 2.2. RNA sequencing and bioinformatics analysis

Total RNA was extracted from the isolated mesothelial cells using a commercial kit. RNA quality was assessed using the Agilent 2100 Bioanalyzer, and only samples with an RNA Integrity Number (RIN) greater than 6 were used for library preparation. Ribosomal RNA was depleted to enrich for mRNA. Sequencing libraries were constructed using a standard protocol involving mRNA fragmentation, reverse transcription, end repair, poly-A tailing, and adapter ligation. The prepared libraries were then sequenced on an Illumina platform.

For bioinformatic analysis, raw sequencing data were processed to identify differentially expressed genes (DEGs) between the long-term and short-term PD groups. Genes with a log2 fold change (logFC) > 1 and an adjusted p-value (adj. P) < 0.05 were considered statistically significant. Functional enrichment analysis of the DEGs was performed to identify potential pathways involved in peritoneal fibrosis.

### 2.3. Structure-based virtual screening

To identify novel inhibitors targeting BRD9, a structure-based virtual screening was performed. The workflow was conducted as follows:

1. Compound Library Preparation: A total of 3,447 compounds were selected from the ‘FDA-approved drug’ subset of the ZINC database (https://zinc.docking.org/). This strategy focused on drugs with established clinical use, thereby facilitating potential drug repurposing. The initial 3D structures of the compounds were obtained in mol2 format.
2. Ligand and Protein Preparation: The molecular structures of all compounds were converted to pdbqt format using Open Babel (version 2.3.2). Preparation included the addition of hydrogen atoms, assignment of Gasteiger charges, and energy minimization. The three-dimensional crystal structure of the BRD9 bromodomain (PDB ID: 4NQN) was retrieved from the Protein Data Bank. Using AutoDock Tools, the protein structure was prepared by removing water molecules, adding hydrogens, assigning Kollman charges, and defining the protein as a rigid body.
3. Molecular Docking: Molecular docking of all 3,447 compounds against BRD9 was performed using AutoDock Vina (version 1.5.7). The docking grid box was centered on the co-crystallized ligand’s binding site and was set to a size of 20 Å × 20 Å × 20 Å to sufficiently encompass the entire binding pocket. The exhaustiveness parameter was set to 20 to ensure an adequate conformational search.
4. Result Analysis and Lead Compound Selection: Following the docking simulation, all compounds were ranked based on their Vina docking score (predicted binding free energy in kcal/mol). The top five compounds with the lowest (most favorable) binding free energies were selected as lead candidates for subsequent experimental validation (Table 1).

**Table 1.** Top 5 compound.

Compound	Affinity (kcal/mol)	Structure	IC_50_ (μM)
Dolutegravir	−8.624	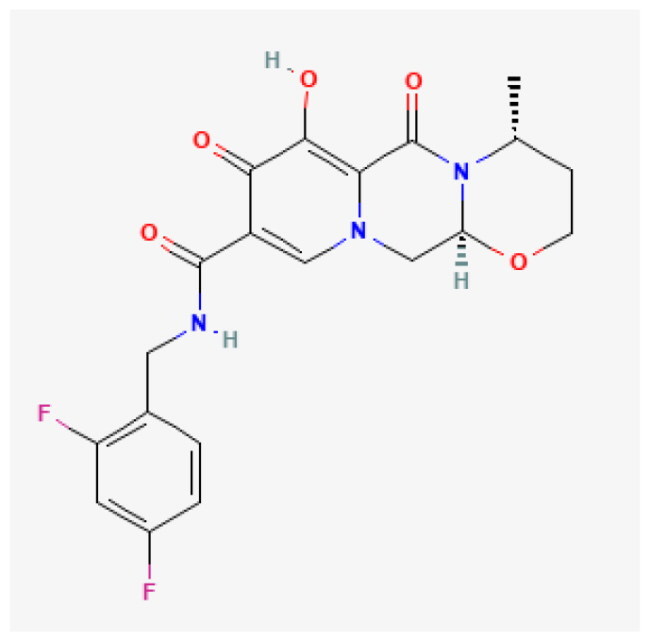	5.28
Vorapaxar	−8.544	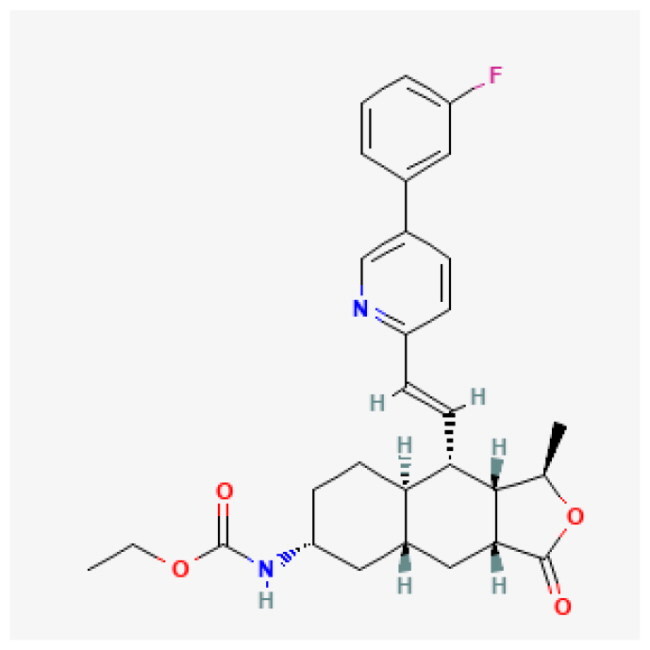	13.11
Lumacaftor	−8.371	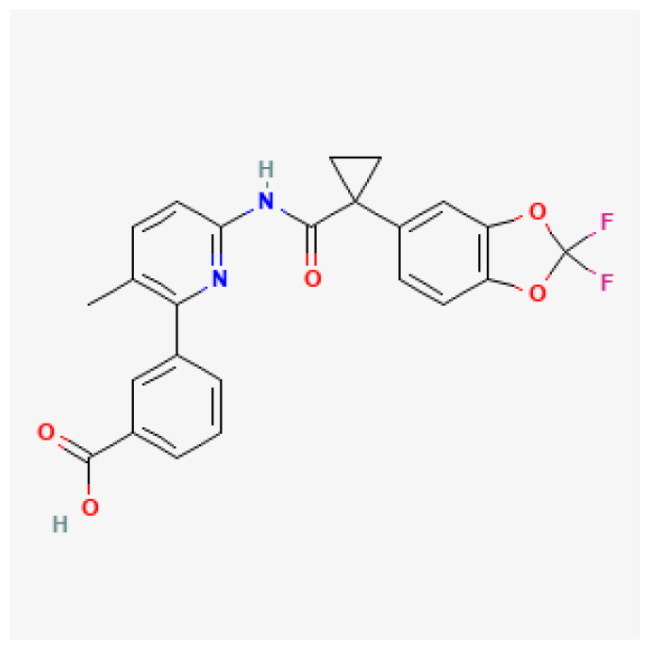	80.48
Hydroxytolbutamide	−8.234	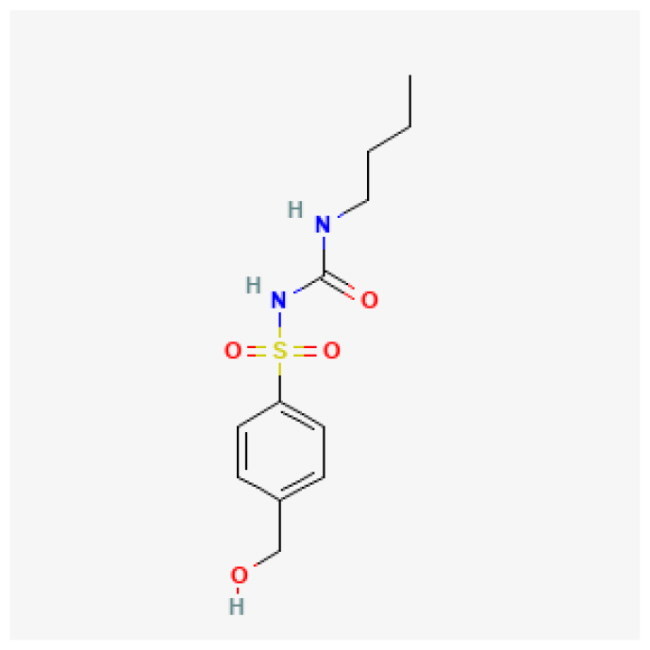	381.30
Enalaprilat	−8.201	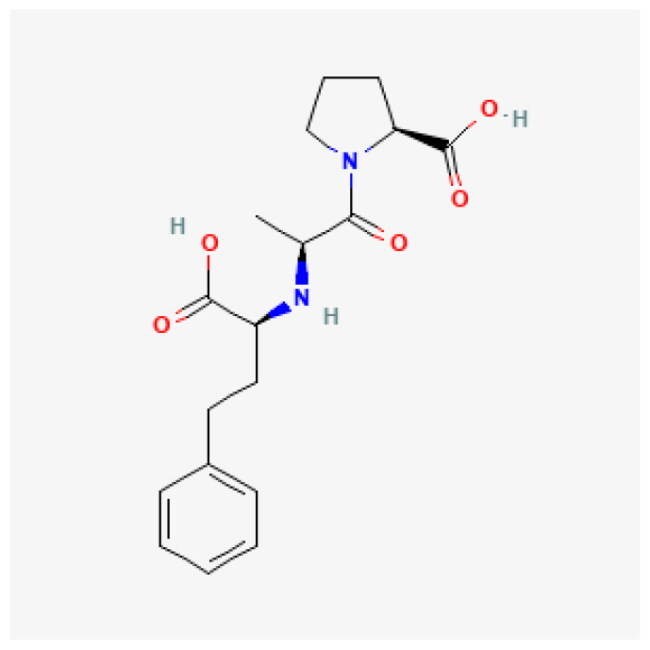	10.89

### 2.4. Visualization

To better understand the binding interactions, three-dimensional (3D) visualization was conducted using PyMOL (version 1.74), a widely used molecular graphics tool. PyMOL enabled detailed analysis of hydrogen bonding and bond distances between the protein and ligands, offering clear insight into the spatial configuration of atomic interactions.

### 2.5. Cell culture and treatments

Human peritoneal fibroblasts were obtained from Yizefeng Biotechnology Company. The human peritoneal mesothelial cell line HMrSV5 was purchased from Shanghai Aoruicell Biological Company. HMrSV5 cells were cultured in high-glucose Dulbecco’s Modified Eagle Medium (H-DMEM; YuanPei, L110KJ). The medium was supplemented with 10% fetal bovine serum (FBS; Gibco, 10099-141), which provides essential nutrients, growth factors, and hormones required for cell growth and proliferation. Human peritoneal fibroblasts were maintained in a fibroblast-specific culture medium (Yizefeng, 2301). These cells were incubated at 37 °C in a 5% CO_2_ atmosphere. HMrSV5 Cells were treated with the corresponding inhibitors according to the experimental groups.

The control group served as a negative control, while the high glucose (HG) group acted as a high-glucose model group, where cells were cultured in 4.25% high-glucose peritoneal dialysis fluid for 48 h. In the HG + benazepril group, cells were treated with 10 μM benazepril (positive control) for 48 h under HG conditions [18]. For the HG + BRD9 inhibitor groups, HG-induced cells were treated with dolutegravir at concentrations of 0.5 μM (low dose, HG + inh LD), 1.5 μM (medium dose, HG + inh MD), and 2.5 μM (high dose, HG + inh HD) for 48 h.

### 2.6. CCK-8 assay

The Cell Counting Kit-8 (CCK-8; Beyotime, C0038) was used to determine the half-maximal inhibitory concentration (IC_50_) and to assess cell proliferation. For the determination of IC_50_, after the cells were treated with inhibitors of different concentrations for the corresponding time, CCK-8 reagent was added and incubated for 1 h. The OD value was measured, and the cell survival rate at each concentration was calculated. Based on this, the dose-response curve was fitted and the IC50 value was obtained.

To evaluate proliferation, the cells were grouped and treated, and CCK-8 detection was performed at 0 h and 24 h respectively. Add 10 μL of CCK-8 solution to each well to avoid bubble formation. Incubate in a cell culture incubator for 1 h. Then, directly measure the absorbance value of each well at a wavelength of 450 nm using an enzyme-linked immunosorbent assay reader. Based on this, analyze the changes in cell viability and proliferation at different time points.

### 2.7. Western blotting assay

The binding of dolutegravir to BRD9 was evaluated using the Cellular Thermal Shift Assay (CETSA) combined with Western blotting. HMrSV5 cells were treated with 5 μM dolutegravir or an equivalent volume of DMSO control for 24 h. After treatment, cells were collected, washed with PBS, and resuspended in a protein stability buffer. The cell suspensions were aliquoted into PCR tubes and subjected to a temperature gradient—specifically, 37 °C, 45 °C, 53 °C, 61 °C, 69 °C, and 73 °C—for 5 min using a thermal cycler to induce progressive protein denaturation. Following heating, samples were immediately transferred to ice for 3 min to halt the denaturation process. The cells were then lysed, and soluble proteins were extracted for subsequent Western blot analysis to determine the remaining intact BRD9 protein levels at each temperature. Subsequently, the total proteins of each group of cells were extracted. After digestion with trypsin, the cells were centrifuged at 300 g and 4 °C for 5 min. They were washed twice with pre-cooled PBS (Beyotime, C0221A), lysed with RIPA lysis buffer containing PMSF, and then centrifuged at 12,000 g and 4 °C for 5 min. The supernatant was collected and stored at −80 °C. The protein concentration was determined by the BCA method (Beyotime, P0012). The samples were boiled in 5 × loading buffer for 10 min and stored at −20 °C for future use.

The protein samples were subjected to SDS-PAGE gel electrophoresis (80 V for 30 min, followed by 120 V electrophoresis), then transferred to methanol-activated PVDF membrane. The membranes were blocked with 5% BSA/TBST for 1 h. Subsequently, it was incubated overnight with the primary antibody at 4 °C (all diluted 1:1000): NLRP3 (263899), Caspase-1 (ab179515), GSDMD (210070), GSDMD-N (ab215203), smooth muscle alpha-actin 2 (ACTA2) (ab124964), Collagen type I alpha 1 (COL1A1) (ab316222), and β-actin (ab8227) as the internal reference. After washing, membranes were incubated at room temperature with the corresponding secondary antibody (1:1000) for 1 h, and develop using a chemiluminescence system. The relative expression level of the target protein was calculated by Image Pro Plus 6.0 software and expressed as the gray value ratio of the target protein to the internal reference β-actin, thereby evaluating the effect of dolutegravir binding on the thermal stability of the target protein and its expression changes in downstream signaling pathways.

### 2.8. Real-time quantitative reverse transcription PCR (RT-qPCR)

Total RNA extraction from cells was performed using TRIzol^™^ Reagent (TAKARA, 740404, Japan) [19]. cDNA synthesis was performed using PrimeScript^™^ RT Master Mix (Yeasen, 11156ES60). The amplification reaction was carried out on a real-time fluorescent quantitative PCR instrument (Agilent, Stratagene Mx3000P) using SYBR^®^ Green Pro Taq HS premix qPCR reagent (Yeasen, 11189ES08, China). The primer sequences included H-β-actin (5′-CATGTACGTTGCTATCCAGGC-3′ and 3′-CTCCTTAATGTCACGCACGAT-5′), H-NLRP3 (5′-TCACCATGTGCTTCATCCCC-3′ and 3′-GGTGGTCTTGGATGTCTGGG-5′), H-ACTA2 (5′-ACTGCTGAGCGTGAGATTGTC-3′ and 3′-GATGCTGTTGTAGGTGGTTTCA-5′) and H-COL1A1 (5′-GCCACGACAAAGCAGAAACA-3′ and 3′-AACAGAACAGTCTCTCCCGC-5′).

### 2.9. RNA stability detection

After grouping and culturing the cells as required, each group of cells was treated with actinomycin D (Medchemexpress, HY-17559) at a final concentration of 2 μg/mL. The treatment durations were set at 0, 1, 3, and 6 h to assess RNA stability over time. At the end of each treatment period, cells from each group were harvested for RT-qPCR.

### 2.10. Flow cytometry

Cell apoptosis was assessed using Annexin V-FITC/PI double staining. After treatment, cells were trypsinized, centrifuged (300 ×g, 4 °C, 5 min), and washed twice with ice-cold PBS under the same centrifugation conditions. Approximately 2.5 × 10^5^ cells were resuspended in 100 µL of 1× Binding Buffer (Beyotime, C1062M), followed by the addition of 5 µL Annexin V-FITC (Beyotime, C1062M) and 10 µL PI staining solution (Beyotime, C1062M). After incubation at room temperature in the dark for 15 min, 400 µL of 1× Binding Buffer was added and mixed gently. Samples were kept on ice and analyzed within 1 h using a flow cytometer (Beckman, DxFlex). First, the cell clusters are delineated in the FSC-A/SSC-A scatter plot. Then, adherent cells are excluded using FSC-H/FSC-A. In the FITC-A/PI-A dual-parameter scatter plot, analysis is conducted as follows: cells with single positive Annexin V-FITC (Q4 quadrant) are identified as early apoptotic cells, and cells with double positive Annexin V-FITC and PI (Q2 quadrant) are identified as late apoptotic/necrotic cells.

Pyroptosis was evaluated by FLICA/PI double staining. Cells were collected and washed as described above. Then, 2.5 × 10^5^ cells were resuspended in 290 µL medium, followed by the addition of 10 µL 30× FLICA reagent (ImmunoChemistry, ICT091) and 1.5 µL PI staining solution. After 30 min of incubation at room temperature in the dark, cells were washed twice with 1× Apoptosis Wash Buffer (ImmunoChemistry, ICT091) and finally resuspended in an appropriate volume of buffer. Analysis was performed within 1 h using a flow cytometer (Beckman, DxFlex). The steps of cell cluster delineation and exclusion of adherent bodies are the same as those in apoptosis analysis. The analysis is conducted in the corresponding fluorescence channels (FLICA/PI) dual-parameter scatter plots: cells with single positive FLICA (Q4 quadrant) represent cells with caspase-1 activity, and cells with double positive FLICA and PI (Q2 quadrant) are determined as cells undergoing pyroptosis.

### 2.11. Immunofluorescence

Adherent cells were seeded on polylysine-coated coverslips. When cells reached approximately 80% confluency, they were washed twice with PBS. Suspension cells in the logarithmic growth phase were harvested by centrifugation (1000 rpm, 5 min) and washed twice with PBS. Cell smears were prepared using a cytocentrifuge or direct smearing. Cell samples were fixed with 4% paraformaldehyde (Beyotime, P0099) for 15 min, permeabilized with 0.1% Triton X-100 (Beyotime, ST795) for 5–15 min, and rinsed three times with PBS (5 min each). Subsequently, samples were blocked with 5% BSA (Solabao, A8020) in PBS for 30 min. After blocking, samples were incubated with the primary antibody Anti-GSDMD-N (ab215203, Abcam) for 1 h at room temperature or overnight at 4 °C. Following three 5-min washes with PBST, a FITC-conjugated secondary antibody (goat anti-rabbit IgG, Beyotime, A0562; 1:500 dilution) was applied and incubated for 1 h at room temperature in the dark. After three additional PBST washes and a brief rinse with distilled water, coverslips were mounted using an anti-fade mounting medium containing DAPI (Beyotime, P0131). Images were acquired using a fluorescence microscope (Zeiss), and the expression and subcellular localization of GSDMD-N protein were analyzed based on fluorescence signals.

### 2.12. Enzyme-linked immunosorbent assays (ELISA)

Cell culture supernatant was collected and centrifuged at 1000 ×g for 10 min to remove cells and debris. Standards provided in the kit were reconstituted and serially diluted according to the manufacturer’s instructions to generate a standard curve. Samples and standards were added to a precoated 96-well plate. Following incubation with detection antibodies specific for IL-1β (Solarbio, SEKH-0002), IL-18 (Solarbio, SEKH-0028), or TGF-β1 (Solarbio, SEKH-0316) at room temperature for the designated time, streptavidin-HRP and substrate solution were added sequentially, with appropriate incubation steps between each addition. The reaction was terminated using stop solution. The optical density was measured at the appropriate wavelength using a microplate reader, and cytokine concentrations were calculated based on the standard curve.

### 2.13. Statistical analyses

The data were analyzed and graphed using Graphpad Prism 9.5.0. All data were expressed as means ± SD. Two-way ANOVA and one-way ANOVA tests were used for inter-group statistics. A P value less than 0.05 was considered a significant difference.

## 3. Results

### 3.1. Virtual screening identifies dolutegravir as a potent BRD9 inhibitor in peritoneal fibrosis

**Figure 1.**
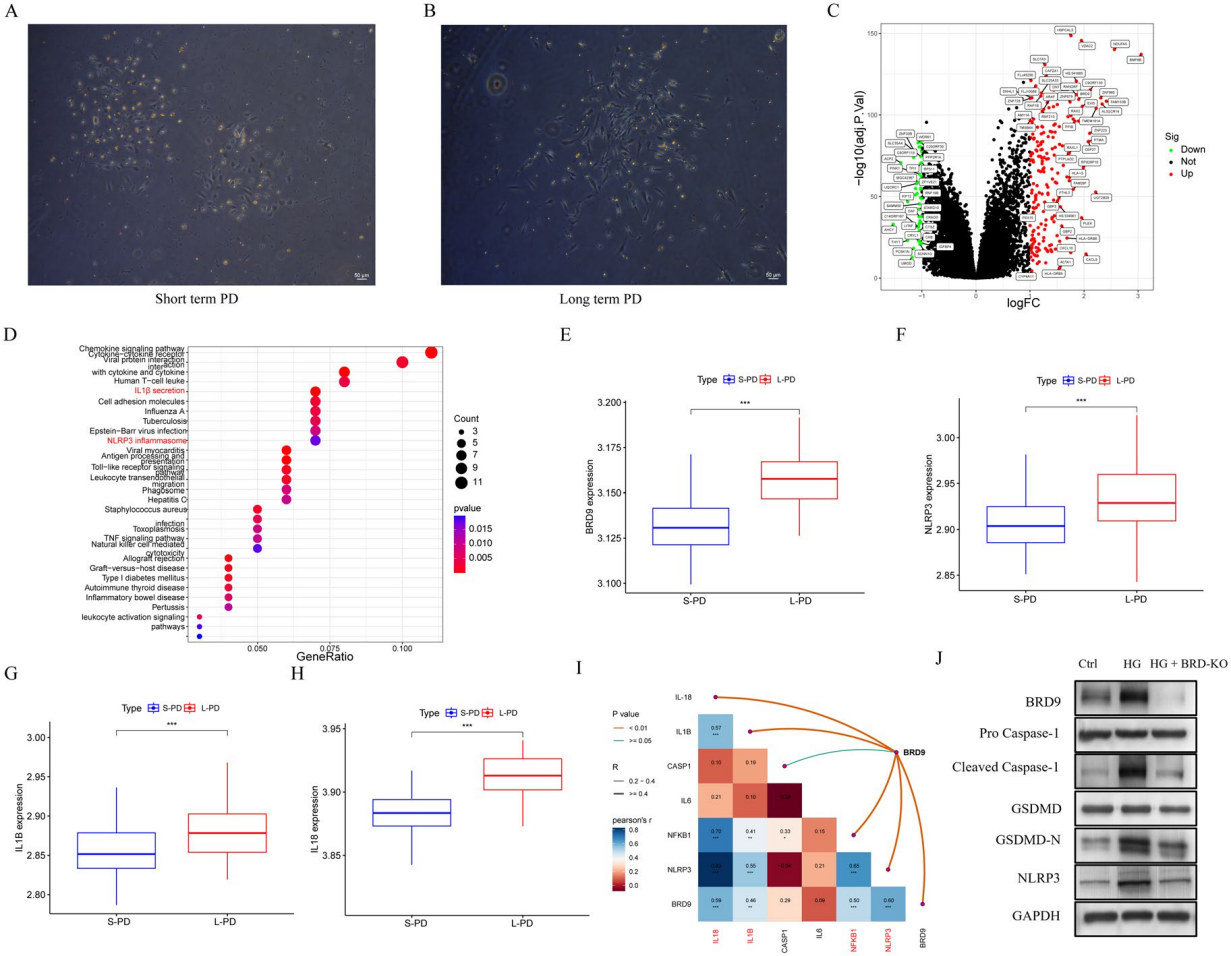
BRD9 is Upregulated and Correlates with Pyroptosis in Peritoneal Fibrosis. (A, B) Representative phase-contrast micrographs showing the typical cobblestone morphology of human peritoneal mesothelial cells isolated and cultured from peritoneal dialysis effluent. Scale bar, 100 μm. (C) Volcano plot of transcriptomic data from mesothelial cells of short-term (n = 9) versus long-term (n = 11) peritoneal dialysis (PD) patients. Genes with significantly differential expression (logFC > 1, adj. P < 0.05) are highlighted in red (upregulated) and blue (downregulated). (D) KEGG pathway enrichment analysis of the differentially expressed genes from (C). The NOD-like receptor signaling pathway (pyroptosis-related) is highlighted. (E-H) mRNA expression levels of pyroptosis-related molecules (BRD9 (E), NLRP3 (F), IL-1β(G)) and IL-18 (H) in mesothelial cells from short-term and long-term PD patients. Data are presented as mean ± SEM (***P < 0.001). (I) Correlation analysis between BRD9 mRNA expression and the expression of NLRP3, IL-1β, and IL-18 in patient-derived mesothelial cells. Pearson correlation coefficients (r) and P-values are indicated.

Mesothelial cells were successfully isolated and cultured from peritoneal dialysis effluent of enrolled patients. The isolated cells exhibited a characteristic cobblestone morphology under microscopy, which is typical of human peritoneal mesothelial cells (Figure 1A,B). To investigate the molecular alterations associated with long-term peritoneal dialysis, we performed RNA sequencing on these cells. Volcano plot analysis of the transcriptome data revealed a distinct profile of differentially expressed genes between the short-term and long-term PD groups (Figure 1C). Notably, among these, BRD9 was significantly upregulated, drawing our attention to its potential role. KEGG pathway enrichment analysis further indicated that these genes were significantly involved in critical processes such as the NLRP3 signaling pathway and IL-1β pathways (Figure 1D). Analysis of the transcriptome data demonstrated that the expression levels of key pyroptosis-related molecules, including NLRP3, IL-1β, and IL-18, were significantly elevated in mesothelial cells from long-term PD patients compared to the short-term group (Figure 1E–G). Concurrently, the expression of BRD9 was also markedly upregulated in the long-term PD group (Figure 1H). Furthermore, a strong positive correlation was observed between the mRNA expression of BRD9 and the levels of NLRP3, IL-1β, and IL-18 (Figure 1I), suggesting a potential association between BRD9 and the pyroptosis signaling pathway in the context of peritoneal fibrosis. To functionally validate the role of BRD9, we examined its impact on pyroptosis in an *in vitro* model. Exposure of mesothelial cells to high glucose (HG, 4.25%) significantly increased the protein expression levels of NLRP3, cleaved Caspase-1, and the active N-terminal fragment of GSDMD (GSDMD-N) (Figure 1J).

Given this mechanistic insight, we performed structure-based virtual screening to identify potential BRD9 inhibitors. A total of 3,447 compounds from FDA-approved (ZINC15) and DrugBank libraries were docked against the bromodomain of BRD9 (PDB: 4NQN) using AutoDock Vina. Compounds were ranked by binding affinity (kcal/mol), and the top five candidate compounds are shown in Table 1.

### 3.2. Inhibitory potency of inhibitors on HMrSV5 cells and dolutegravir’s impact on BRD9 protein stability

**Figure 2.**
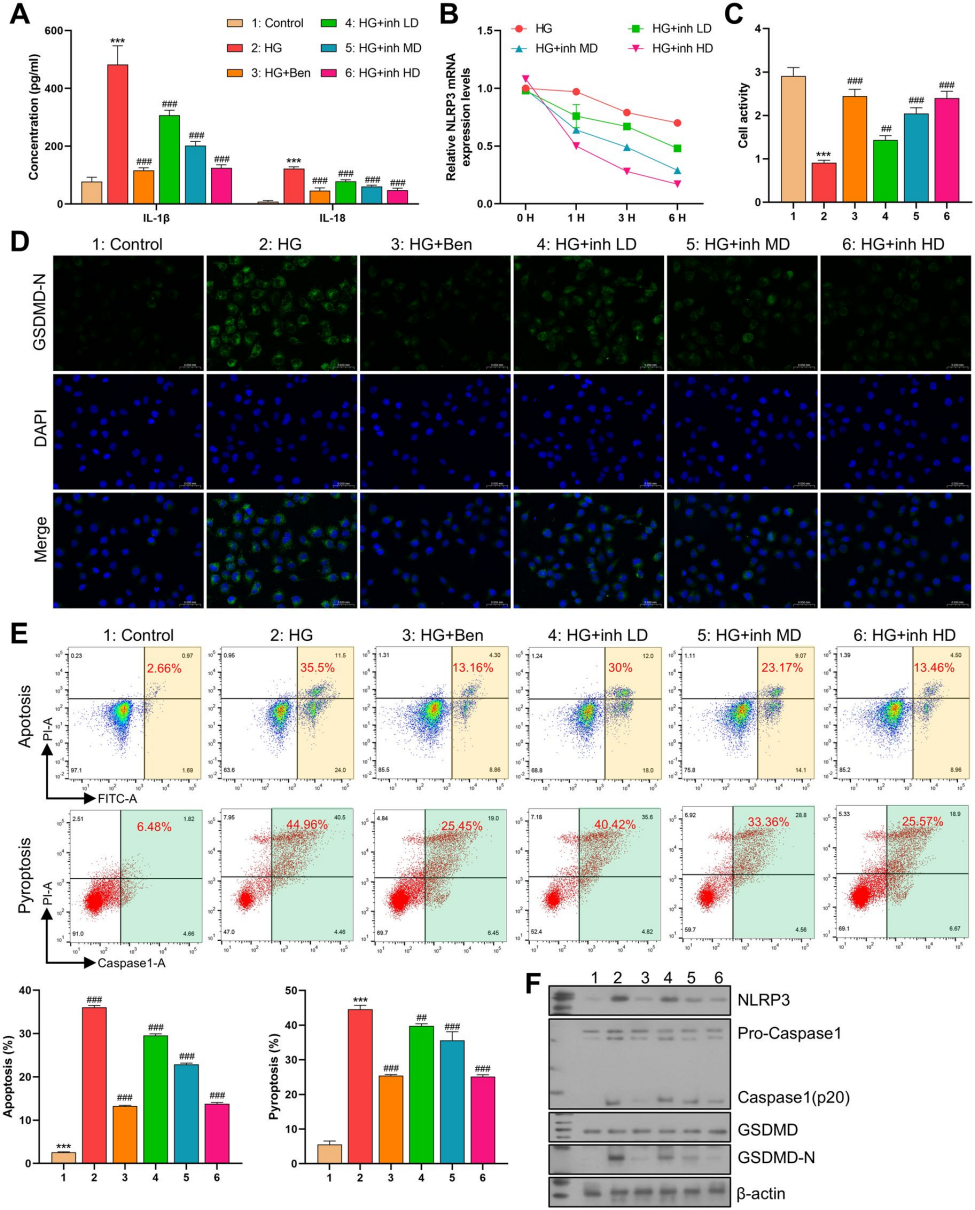
Dolutegravir Exhibits Superior BRD9-Targeted Inhibition: From Cytotoxicity Profiling to Structural Validation. (A) Determine the IC_50_ of five drugs on the peritoneal mesothelial cells of HMrSV5. dolutegravir: IC_50_ = 5.277 μM; Vorapaxar: IC_50_ = 13.11 μM; Lumacaftor: IC50 = 80.48 μM; Hydroxytolbutamide: IC_50_ = 381.3 μM; Ena April at: IC_50_ = 10.89 μM. (*n* = 3) (B) After incubating 5 μM dolutegravir with HMrSV5 peritoneal mesothelial cells for 24 h, the CETSA-WB assay confirmed the interaction between dolutegravir and BRD9 protein. (*n* = 3) (C) Pymol visualizes the connection between dolutegravir and BRD9 protein.

We evaluated the inhibitory effects of five inhibitors on HMrSV5 peritoneal mesothelial cells using the CCK − 8 assay. The half-maximal inhibitory concentration (IC50) values were calculated for each inhibitor. Among them, dolutegravir exhibited the lowest IC50 value, indicating its superior inhibitory potency on HMrSV5 cells compared to the other four inhibitors (Figure 2A). Based on these results, dolutegravir was selected for subsequent experiments. To further investigate the effect of dolutegravir on protein stability, we conducted a cellular thermal shift assay (CETSA). The results demonstrated that, in comparison with the DMSO-treated control group, dolutegravir significantly reduced the degradation rate of the BRD9 protein (Figure 2B). This finding suggests that dolutegravir may interact with BRD9, thereby influencing its thermal stability and potentially its function within the cells. Figure 2C shows the conformations of BRD9 and dolutegravir.

### 3.3. BRD9 inhibitor’s impact on pyroptosis pathway in peritoneal fibrosis-related cells

**Figure 3.**
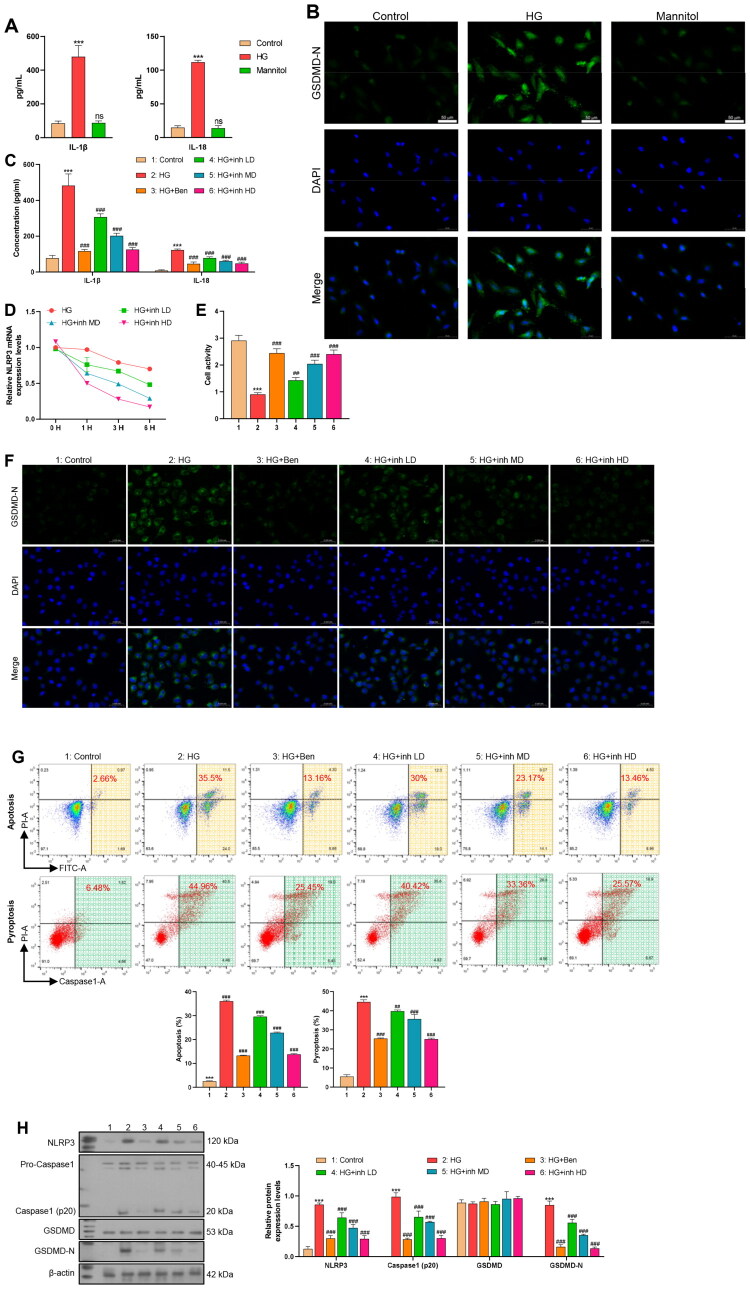
Dolutegravir regulates NLRP3 to inhibit pyroptosis of mesothelial cells. (A) ELISA detection of the expression levels of IL-1β and IL-18 in the cell supernatants of each group. (*n* = 3) (B) Immunofluorescence was used to detect the expression level of GSDMD-N in cells of each group (green: GSDMD-N; blue: nuclear). (*n* = 3) (C) ELISA detection of the expression levels of IL-1β and IL-18 in the cell supernatants of each group. (*n* = 3) (D) After treating cells with actinomycin D (2 μg/mL), the stability of NLRP3 mRNA was detected by qPCR. (*n* = 3) (E) CCK8 was used to detect the cell viability of each group. (*n* = 3) (F) Immunofluorescence was used to detect the expression level of GSDMD-N in cells of each group (green: GSDMD-N; blue: nuclear). (*n* = 3) (G) The proportion of apoptosis and pyroptosis in each group was detected by flow cytometry. (*n* = 3) (H) WB was used to detect the protein expression levels of NLRP3, Caspase1, GSDMD, and GSDMD-N in each group of cells. (*n* = 3) (*: vs.Control; #: vs.HG).

In this study, we established multiple experimental groups to investigate the effects of BRD9 inhibitors on peritoneal fibrosis-related cell pyroptosis pathways. Firstly, to eliminate the confounding effects of hyperosmotic stress, additional experiments confirmed that isotonic mannitol treatment did not trigger a significant pyroptosis response (Figure 3A,B). This clarified that the subsequent effect was specifically related to high glucose. ELISA results showed that the levels of inflammatory cytokines IL-1β and IL-18 in the HG group were significantly higher than those in the control group. Meanwhile, treatment with the BRD9 inhibitor dolutegravir significantly reduced the high glucose-induced secretion of inflammatory cytokines, to an extent comparable to the positive control (Figure 3C). CCK-8 assay and flow cytometry showed that the cell viability was the lowest in the HG group. In contrast, BRD9 inhibitors could inhibit apoptosis and pyroptosis, thereby promoting cell survival (Figure 3E,G). Western blot analysis showed that the expression levels of NLRP3, Caspase-1, and GSDMD-N were significantly higher in HG group, followed by a decreasing trend with the application of BRD9 inhibitors. The expression of Pro – Caspase-1 and GSDMD remained relatively stable across all experimental groups (Figure 3H). Immunofluorescence analysis of GSDMD-N also showed a similar pattern (Figure 3F). Additionally, qPCR assessment of NLRP3 stability in the actinomycin D experiment demonstrated a decreasing order of NLRP3 stability: HG group > HG + inh LD > HG + inh MD > HG + inh HD (Figure 3D).

### 3.4. High-glucose-induced fibrotic changes and BRD9 inhibitor intervention

**Figure 4.**
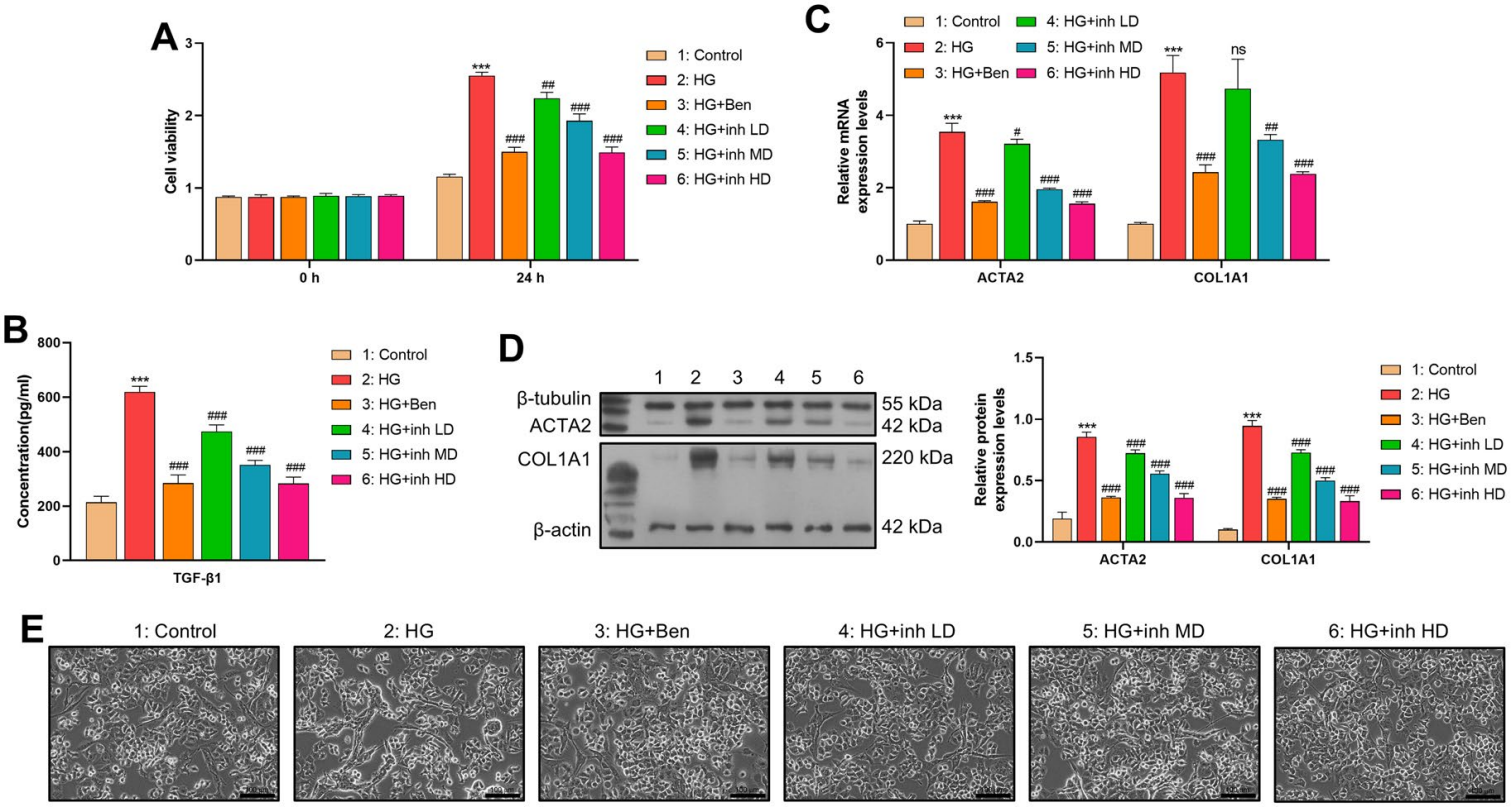
Mesothelial cells promote the profibrotic activation of peritoneal fibroblasts in a co-culture system. (A) CCK8 detection of proliferation of human peritoneal fibroblasts in each group in the co-culture system. (*n* = 3) (B) ELISA was used to detect the expression level of TGF-β1 in the supernatants of each group in the co-culture system. (*n* = 3) (C) qPCR was used to detect the mRNA expression levels of ACTA2 and COL1A1 in human peritoneal fibroblasts of each group in the co-culture system. (*n* = 3) (D) WB was used to detect the protein expression levels of ACTA2 and COL1A1 in human peritoneal fibroblasts of each group after co-culture. (*n* = 3) (E) Cell images of human peritoneal fibroblasts in each group of the co-culture system were captured under an inverted microscope. (*n* = 3) (*: vs.Control; #: vs.HG).

In this study, we conducted a 24-h co-culture of HMrSV5 cells with fibroblasts to investigate the impact of HMrSV5 cells on fibroblasts. Cell proliferation rates varied among the different experimental groups. The order of proliferation, from highest to lowest, was as follows: the HG group > the HG + inh LD group > the HG + inh MD group > the HG + inh HD group = the HG + benazepril group > the control group (Figure 4A). The expression levels of α-SMA and collagen I, two key markers of fibrosis, were quantified using both Western blotting and qPCR (Figure 4C,D). Consistent results were obtained from both methods, revealing that the HG group exhibited the highest expression levels of α-SMA and collagen I. In contrast, treatment with BRD9 inhibitors resulted in a dose-dependent reduction in the expression of these fibrotic markers. The level of TGF-β1 in cell supernatant was detected by ELISA. The changing trend of TGF-β1 levels among different groups was similar to that of α-SMA and collagen I. Among them, the TGF-β1 level was the highest in the HG group, and BRD9 inhibitors reduced the secretion of TGF-β1 by cells (Figure 4B). Under normal physiological conditions, fibroblasts typically exhibit a flat, polygonal morphology, characterized by well-defined cell boundaries and an orderly arrangement. However, when fibroblasts were co-cultured with HMrSV5 cells under high-glucose conditions, significant morphological alterations were observed. Specifically, fibroblasts in the HG group gradually elongated into spindle-shaped cells, displaying features reminiscent of fibrotic cells. Notably, treatment with BRD9 inhibitors was found to ameliorate these fibrotic morphological changes (Figure 4E).

## 4. Discussion

This study establishes dolutegravir—an FDA-approved HIV integrase inhibitor—as a novel BRD9-targeted inhibitor that effectively suppresses high-glucose-induced mesothelial cell pyroptosis and subsequent fibroblast-mediated fibrosis. Our findings not only validate a robust virtual screening (VS) strategy for drug repurposing but also unveil a mechanistic link between pyroptotic cell death and fibrotic signaling in peritoneal injury, offering new therapeutic strategies for PF.

The VS pipeline employed in this study, which leverages FDA-and DrugBank-approved compound libraries, demonstrates high computational efficiency in inhibitor discovery. By utilizing molecular docking and binding free energy prediction, this approach rapidly identified dolutegravir as a candidate BRD9 inhibitor from a pool of 3,447 compounds, significantly improving the efficiency of drug discovery while reducing the resource demands associated with traditional screening methods. CETSA and molecular docking results corroborated that dolutegravir directly binds to BRD9 and stabilizes its protein conformation. This discovery is highly consistent with the research trend in recent years on allosteric sites and their targeting strategies in bromine domain proteins [20]. The cell viability assay determined that the half-inhibitory concentration was within the micromolar range, which provided a suitable nontoxic concentration for subsequent functional experiments.

Our data clearly demonstrate that dolutegravir suppresses pyroptosis in mesothelial cells under high-glucose (HG) conditions—a critical microenvironment in peritoneal dialysis. Mechanistically, it acts by preventing a key step in the pyroptotic cascade: the proteolytic cleavage of caspase-1 and its downstream target GSDMD. This role of BRD9 in regulating inflammation is consistent with its function as a component of the SWI/SNF chromatin remodeling complex, which is pivotal for modulating inflammatory gene expression [21]. Its inhibition by dolutegravir likely impedes NLRP3 inflammasome priming, a known pyroptosis trigger [22]. Our actinomycin D-based qPCR assays revealed a critical dimension of BRD9’s function: it governs NLRP3 mRNA stability in mesothelial cells. Under HG conditions, BRD9 inhibition by dolutegravir accelerated NLRP3 mRNA decay (evidenced by reduced transcript half-life), directly linking epigenetic regulation to inflammasome component turnover [23]. This finding indicates that BRD9 regulates pyroptosis through a two-tiered control of NLRP3 expression. At the transcriptional level, BRD9, *via* the SWI/SNF chromatin-remodeling complex, enhances chromatin accessibility at the NLRP3 promoter to facilitate its transcriptional activation [21,24]. At the post-transcriptional level, BRD9 further sustains NLRP3 mRNA stability, potentially by recruiting RNA-binding proteins such as HuR to delay mRNA degradation and by suppressing destabilizing microRNAs including miR-223. This hypothesis is based on the known function of HuR in stabilizing pro-inflammatory mRNAs and the reported literature that miR-223 can target and regulate NLRP3 [25,26]. Verifying this hypothesis will be an important direction for future research, and it can be explored through techniques such as RNA immunoprecipitation (RIP), CLIP-seq, or microRNA omics, combined with functional restoration experiments to uncover the precise mechanism of BRD9’s post-transcriptional regulation of NLRP3 mRNA.

Crucially, we explain how mesothelial cell pyroptosis leads to fibroblast fibrosis. This study, from the perspective of intercellular communication, clarified how pyroptosis of mesothelial cells promotes the fibrosis process. Mesenchymal cells with pyroptosis release a large amount of IL-1β, IL-18 and the key pro-fibrotic factor TGF-β1. These mediators activate adjacent fibroblasts through paracrine action, induce their proliferation and differentiation into myofibroblasts, and promote the excessive deposition of extracellular matrices such as collagen [27]. Dolutegravir effectively cuts off this powerful paracrine pro-fibrotic signal by inhibiting pyroptosis of mesothelial cells from the source, thereby blocking the subsequent fibrotic cascade reaction. This discovery not only reveals an intercellular dialogue pathway that is crucial for the development of peritoneal fibrosis, but also is highly consistent with the recent view that inflammasome activation is regarded as a core driver of fibrotic diseases [28]. We emphasize that this model remains a testable hypothesis. Its validity requires future verification through definitive genetic experiments—specifically, loss-of-function and gain-of-function studies targeting BRD9—to confirm whether the anti-fibrotic effects of dolutegravir are contingent upon BRD9 inhibition.

Although this study initially revealed the role and mechanism of dolutegravir as a BRD9 inhibitor in anti-peritoneal fibrosis, there are still several limitations. Firstly, the binding pockets of bromodomain family members (such as BRD4, BRD7, BRD9) are highly similar, which poses inherent challenges for the development of highly selective inhibitors. Although molecular docking, CETSA, and functional experiments all support the interaction between dolutegravir and BRD9, we have not systematically evaluated its potential off-target effects on other family members. Recent studies have made progress in addressing this issue by identifying BRD9-specific conformational switches (such as the flipping of Phe163) [29]. Therefore, we cautiously believe that the anti-fibrotic effect of this study may mainly be mediated by the inhibition of BRD9, but it cannot be completely ruled out that it may also exert its effect by influencing structurally similar bromodomain proteins. The exact mode of the combination of dolutegravir and BRD9 still needs to be further verified through structural biology methods such as X-ray crystallography or Cryo-EM [30]. Secondly, all experiments were conducted in cell models. The lack of *in vivo* experimental data limits our understanding of the efficacy and safety of dolutegravir in complex physiological environments. Future research urgently needs to be conducted in animal models, for example, establishing a mouse model of chronic peritoneal dialysis, inducing peritoneal fibrosis through long-term intraperitoneal injection of high-sugar dialysis fluid, and then evaluating the effects of dolutegravir (intraperitoneal or systemic administration) on peritoneal function (such as ultrafiltration capacity), peritoneal histological changes (thickness, collagen deposition), as well as the expression of *in situ* pyroptosis and fibrosis markers [31]. In addition, the specific molecular chaperones and pathways by which BRD9 regulates mRNA stability after transcription have not been fully elucidate and need to be further explored through techniques such as RNA co-immunoprecipitation (RIP) and CLIP-seq [32]. Finally, as a marketed drug, the long-term safety of dolutegravir and its pharmacokinetic characteristics in the local peritoneal environment still need to be evaluated. Future work will focus on the above issues, providing a more solid theoretical foundation and experimental basis for targeted BRD9 treatment of peritoneal fibrosis.

Any strategy of repurposing an old drug must carefully weigh its benefits against its risks. One of the known side effects of dolutegravir is reversible elevation of serum creatinine [33], which mainly results from the inhibition of renal tubular creatinine secretion rather than renal glomerular damage. Nevertheless, for patients with end-stage renal disease (especially those undergoing peritoneal dialysis), this pharmacological characteristic still needs to be closely monitored and evaluated in future preclinical and clinical studies to clarify its safety in specific populations. At the same time, within the field, there have emerged highly selective BRD9 inhibitors (such as Sendegobresib in Phase I clinical trials, NCT05355753) and degraders. Compared with these ‘refined’ tool compounds, the selectivity and potency of dolutegravir as a BRD9 inhibitor may be lower, but its advantage lies in having a complete human safety database and clinical accessibility. Therefore, the realistic clinical prospect of repositioning moltelavir for the treatment of peritoneal fibrosis can be regarded as a phased approach: This study initially positions it as an easily accessible tool for rapidly validating the therapeutic hypothesis of ‘inhibiting BRD9-alleviating pyroptosis-reducing peritoneal fibrosis’ in preclinical models. Positive results will significantly reduce the development risks for subsequent development targeting this target; on this basis, if the hypothesis is confirmed, the logical next step is not to directly use moltelavir for clinical use, but to develop optimized derivatives based on its structure, aiming to maintain good pharmacokinetic properties while enhancing the selectivity, potency, and safety profile for BRD9 and tailoring it for use in patients with kidney diseases; ultimately, the specifically designed BRD9 inhibitors produced by this research pipeline will have a clearer regulatory and clinical development path than the parent compound that was directly repositioned, and are expected to provide a treatment option for peritoneal fibrosis based on a new mechanism.

## 5. Conclusion

In conclusion, this study identifies dolutegravir as a previously unrecognized BRD9 inhibitor through structure-based virtual screening of approved drugs and validates its direct binding and inhibitory activity in mesothelial cells. Functionally, BRD9 inhibition by dolutegravir attenuates high-glucose-induced mesothelial pyroptosis by suppressing NLRP3 inflammasome activation and accelerating NLRP3 mRNA decay, thereby limiting caspase-1 and GSDMD cleavage and reducing IL-1β/IL-18 release. By disrupting this pyroptosis-driven paracrine loop, dolutegravir also diminishes TGF-β1 secretion and fibroblast activation, ultimately alleviating pro-fibrotic remodeling. These findings not only delineate a druggable BRD9–NLRP3 axis in peritoneal fibrosis but also support computational drug repurposing as an efficient strategy to discover anti-fibrotic therapies, positioning dolutegravir as a promising candidate for further preclinical and clinical evaluation.

## Data Availability

The data used to support the findings of this study are available from the corresponding author upon reasonable request.
